# Case of Sudden Blindness, (Produced by Effusion of Blood on the Optic Nerve?)

**Published:** 1845-03

**Authors:** 


*Case of sudden blindness, (produced by effusion of blood on the optic nerve?).*—M. D., aged 18, a laundry-maid, of strumous appearance, came to me on the morning of the 8th of October. The previous evening, as she was reaching up to take some clothes off a line, she experienced a sensation as if something had fallen into her left eye, followed by total and instantaneous loss of vision. There was no lachrymation nor any inflammation of the conjunctiva; the cornea and humours perfectly clear and unclouded, so that it was evident there was no effusion of blood within the eyeball. There was slight redness of the parts surrounding the eye, probably produced by the patient's having rubbed the eye a good deal. The iris contracted and dilated actively, according as the light was more or less intense; though there was no perception whatever of light, even on holding a candle pretty close to the eye. On making an examination, I found there was no foreign body present, though she still complained of the sensation of something being in the eye. There was deep-seated pain in the orbit, and supra-orbital headache. She was ordered to take a brisk purgative, to have leeches to the temple, and afterwards to keep a cold lotion applied to the eye. She was further directed, as soon as the medicine had operated freely, to take a powder composed of Calomel, gr. i. $\frac{1}{2}$, Opium, gr. $\frac{1}{3}$, Tartarized Antimony, gr. $\frac{1}{8}$, which was to be repeated every second hour.

The next day a blister was applied to the temple, and the mercury continued. On the 10th the leeching was repeated, as she complained greatly of the pain in the orbit and forehead. Mercury continued.

11th.—Mouth becoming tender. Continued the mercury. In the evening she could distinguish the light of a candle.

12th.—Could see pretty well; the gums sore. Stopped giving mercury.

13th.—Vision quite restored, and has continued so ever since. There was no fever at any time during this attack, and the pulse was never above 70, soft and full.—*Ibid.*

